# New insights into permeability determination by coupling Stoneley wave propagation and conventional petrophysical logs in carbonate oil reservoirs

**DOI:** 10.1038/s41598-022-15869-1

**Published:** 2022-07-08

**Authors:** Alireza Rostami, Ali Kordavani, Shahin Parchekhari, Abdolhossein Hemmati-Sarapardeh, Abbas Helalizadeh

**Affiliations:** 1 Department of Petroleum Engineering, Petroleum University of Technology (PUT), Ahwaz, Iran; 2 Department of Petrophysics Engineering, National Iranian South Oil Company (NISOC), Ahwaz, Iran; 3 Department of Petroleum Engineering, Kish International Campus, University of Tehran, Kish, Iran; 4 Department of Petroleum Engineering, Shahid Bahonar University of Kerman, Kerman, Iran; 5 Key Laboratory of Continental Shale Hydrocarbon Accumulation and Efficient Development, Ministry of Education, Northeast Petroleum University, Daqing, 163318 China

**Keywords:** Solid Earth sciences, Energy science and technology, Engineering

## Abstract

The need to determine permeability at different stages of evaluation, completion, optimization of Enhanced Oil Recovery (EOR) operations, and reservoir modeling and management is reflected. Therefore, various methods with distinct efficiency for the evaluation of permeability have been proposed by engineers and petroleum geologists. The oil industry uses acoustic and Nuclear Magnetic Resonance (NMR) loggings extensively to determine permeability quantitatively. However, because the number of available NMR logs is not enough and there is a significant difficulty in their interpreting and evaluation, the use of acoustic logs to determine the permeability has become very important. Direct, continuous, and in-reservoir condition estimation of permeability is a unique feature of the Stoneley waves analysis as an acoustic technique. In this study, five intelligent mathematical methods, including Adaptive Network-Based Fuzzy Inference System (ANFIS), Least-Square Support Vector Machine (LSSVM), Radial Basis Function Neural Network (RBFNN), Multi-Layer Perceptron Neural Network (MLPNN), and Committee Machine Intelligent System (CMIS), have been performed for calculating permeability in terms of Stoneley and shear waves travel-time, effective porosity, bulk density and lithological data in one of the naturally-fractured and low-porosity carbonate reservoirs located in the Southwest of Iran. Intelligent models have been improved with three popular optimization algorithms, including Coupled Simulated Annealing (CSA), Particle Swarm Optimization (PSO), and Genetic Algorithm (GA). Among the developed models, the CMIS is the most accurate intelligent model for permeability forecast as compared to the core permeability data with a determination coefficient (R^2^) of 0.87 and an average absolute deviation (AAD) of 3.7. Comparing the CMIS method with the NMR techniques (i.e., Timur-Coates and Schlumberger-Doll-Research (SDR)), the superiority of the Stoneley method is demonstrated. With this model, diverse types of fractures in carbonate formations can be easily identified. As a result, it can be claimed that the models presented in this study are of great value to petrophysicists and petroleum engineers working on reservoir simulation and well completion.

## Introduction

Reservoir rock permeability is one of the essential information in oil and gas production. In this way, when exploring and evaluating a hydrocarbon reservoir, permeability is used to develop the field and optimize the completion of wells^1^. According to the permeability parameter, the reservoir boundaries, the contact level of water and oil, and the most suitable perforation interval in the well are determined. In general, several applications can be considered for permeability^1^: optimizing the completion and production of wells to achieve maximum oil production and minimum water cut from considered wells; production forecasting and planning to achieve maximum recover factor from the studied reservoir; and determining the best reservoir drainage pattern and optimal drilling location. While having an absolute value is valuable for reservoir permeability, generating the well permeability profile is also of significant importance. However, achieving a well permeability profile is one of the most challenging engineering measurements.

For this purpose, two direct and indirect methods have been invented in past decades. In the direct method, measurements are made at several points along the well, including well testing techniques, Repeat Formation Tester (RFT), and analysis of core samples taken from wells under different conditions. In the indirect method, permeability can be determined by interpreting various properties such as porosity, processing other logs such as Nuclear Magnetic Resonance (NMR) and geochemical logs, and using mathematical models with simplifying assumptions. Because these models are not accurate, their results are highly uncertain. The Stoneley acoustic wave analysis is the only direct and continuous measurement technique for permeability prediction along wellbores. Although the principles of Stoneley wave measurement have long been known, accurate and reliable measurement of the permeability by this method is still challenging^1^.

At low frequencies, the Stoneley waves become cylindrical, pushing borehole fluid piston-like into the formation. When the Stoneley waves reach the permeable or fractured zones, fluid displacement occurs between the wellbore and the formation. As a result, a decrease in energy level and damping of the wave are expectable, which lead to an increase in the wave slowness time. Permeable regions or fractures have a variety of properties and characteristics that affect Stoneley waves in different ways. In the case of permeable fractures, local and robust interferences create the chest pattern reflections in the Variable Density Log (VDL). Besides, unique methods have been developed by several authors to determine the fractures using the Stoneley wave analysis^2–4^.

In addition, it is very challenging to evaluate permeability in carbonate reservoirs because the factors influencing the permeability in carbonates are often different from those in sandstones. It is usually impossible to determine a good relationship between porosity and permeability in carbonate formations. Each method available in this field has advantages and disadvantages^5^. Numerous researchers have shown that the propagation of an acoustic wave in a well containing fluid is fundamentally different from the propagation of a flat wave in the vicinity of a single interference surface. This difference is due to the formation of different types of waves (i.e., surface and internal waves) in the well environment. An acoustic waveform contains valuable information. The most crucial wave components in a full waveform for measuring permeability are Stoneley and shear waves. The researchers found a relation between the Stoneley wave propagation in the well and the rock permeability^6, 7^. They also showed a perfect match between high-frequency (~ 20 kHz) Stoneley wave energy and phase velocity, and attenuation of low-frequency (~ 1 kHz) Stoneley wave with core permeability^6^. Determining the permeability and how it is distributed is very important in many ways, including designing and completing wells for the successful implementation of Enhanced Oil Recovery (EOR) programs (e.g., water flooding) and simulating the reservoir model for optimizing its management^8^. Stoneley waves, which are classified in the group of guided waves, propagate at the interface between wellbore fluid and formation^9^. The effect of slowing down and dampening the Stoneley wave is a function of the frequency so that increasing the permeability increases the wave attenuation and slowness time. Over the past two decades, much progress has been made in acoustic well logging^1^. This advancement, including measurements of high-quality Stoneley waves and Signal-to-Noise Ratios (SNR), is performed using the Slowness/Time Coherence (STC) technique to process the Stoneley waves. This method is based on the development of the Semblance algorithm by Kimball and Marzetta^10^ for acoustic wave processing.

Numerous researchers worldwide have carried out massive studies on the prediction of the permeability and mobility of geological formations using the Stoneley wave analysis method^7, 11–24^. Some of these studies are in the form of mathematical and analytical modeling, and some others utilized empirical/semi-empirical relationships to estimate the absolute rock permeability. Analytical models require a large number of inputs that may not be available and, or maybe estimated roughly. In addition, advanced mathematics is needed to solve these analytical models, which limits their applications. It should be noted that these complex models use several simplifying assumptions to make simpler their solutions, which leads to cumulative deviations from the real answer. Empirical/semi-empirical methods have also been of great importance in permeability calculations. The simplicity of such techniques is one of the main features of such techniques; however, empirical/semi-empirical methods do not have sufficient accuracy in estimating permeability and predicting its complex trend, especially in carbonate reservoirs with natural fractures. The models described above are usually developed for sandstone reservoirs or water-filled synthetic porous environments. To the best of the authors’ knowledge, the researchers have rarely applied the aforementioned methods for naturally fractured carbonate formations around the globe. Therefore, there is a fundamental need for a new and universal method for calculating permeability in carbonate reservoirs with natural fractures. To answer this problem, powerful artificial intelligence methods can be used. Different studies in the literature have proven the successful application of these methods in various branches of science and engineering, especially geosciences and petroleum/chemical engineering^25–34^. Recently, several pieces of research have been implemented via diverse machine learning strategies to investigate the estimation of permeability via logging data^35, 36^. These methods can be used to learn the behavior of scientific phenomena where there is not sufficient data to predict the desired parameters.

In the current investigation, a comprehensive modeling study was carried out by developing many artificial intelligence approaches, including Adaptive Network-Based Fuzzy Inference System (ANFIS), Least-Square Support Vector Machine (LSSVM), Radial Basis Function Neural Network (RBFNN), Multi-Layer Perceptron Neural Network (MLPNN), and Committee Machine Intelligent System (CMIS), for calculating permeability in terms of Stoneley and shear waves travel-time, effective porosity, bulk density and lithological data in one of the naturally-fractured and low-porosity carbonate reservoirs located in the Southwest of Iran. Three Evolutionary Algorithms (EAs), including Genetic Algorithm (GA), Particle Swarm Optimization (PSO), and Coupled Simulated Annealing (CSA), are combined with the prementioned predictive techniques to optimize the modeling. To the best of the authors’ knowledge, it is the first time in literature that these kinds of modeling strategies are carried out for permeability estimation regarding the Stoneley wave analysis in naturally-fractured and low-porosity carbonate reservoirs. Several statistical analyses and graphical means are used to show the performance of the extended techniques via comparing to NMR permeability estimation methods.

There is a bulk of literature studies focusing on permeability prediction via only conventional petrophysical logs such as Neuron (NPHI), Density (RHOB), and Sonic travel time (DT). Nonetheless, the application of advanced sonic tools such as Dipole Shear Sonic Imager (DSI), which prepares several curves (e.g., shear and Stoneley slowness), has not yet been comprehensively studied. In fact, joint use of fullset logs and advanced array sonic logs such as DSI (e.g., shear and Stoneley slowness) in association with a large number of core data is investigated in this study, which distinguishes this research from the available bulk of literature. To make this study comprehensive, several combinations of predictive and optimization approaches as hybrids are developed. Comparing the best model here (termed as CMIS model) with NMR-derived permeability models is another advantage and superiority of this study over other techniques. At last, it is beneficial to express that this study is investigated in low permeability and Naturally-Fractured Carbonate (NFR) oil reservoirs in Southwest Iran; thereby, it could strongly help to detect semi-filled fractures, micro-fractured, and vuggy media easily due to the anomalies occurring in the behavior of Stoneley wave slowness which propagates in the interface of borehole and formation. Vugs and fractures are the main reasons for large energy dissipations happening in the sound wave propagation. Stoneley waves show this phenomenon in the best manner for fracture detection.

## Data gathering

**Figure 1:**
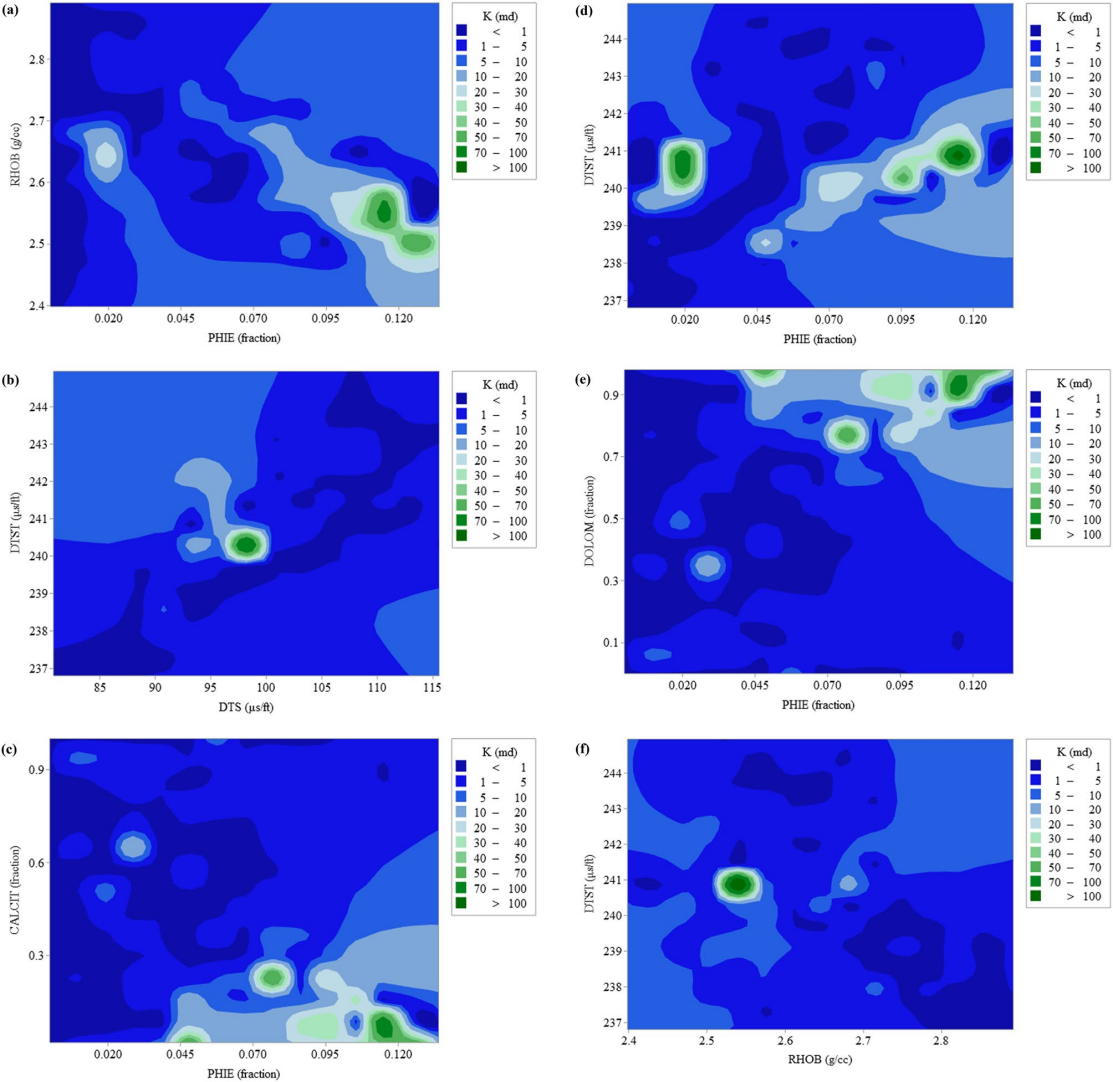
Representation of permeability variation in the low-porosity carbonate reservoir under study: (**a**) permeability vs. bulk formation density and effective porosity, (**b**) permeability vs. Stoneley and shear wave travel times, (**c**) permeability vs. volumetric limestone composition and effective porosity, (**d**) permeability vs. Stoneley wave travel time and effective porosity, (**e**) permeability vs. volumetric dolomite composition and effective porosity, and (**f**) permeability vs. shear wave travel time and bulk formation density.

Collecting a comprehensive database is a prerequisite for robust and accurate modeling^37–41^. For this goal, a complete dataset was first undertaken with an extensive range of variations from the petroleum industry in one of the naturally-fractured and low-porosity carbonate reservoirs. This data set includes Stoneley and shear waves travel-time, effective porosity, bulk density, and lithological data (as modeling inputs), and formation permeability (as modeling output). To prepare this database, the RCAL (Routine Core Analysis) data were depth matched with petrophysical data obtained from logging operations. Approximately 16% of the data bank was allocated for model training. The remaining data (about 84% of the total data bank) was used to check the model's ability to predict permeability in a carbonate formation. This allocation is carried out to include the range of parameters along with the entire reservoir zonation. Figure 1 indicates the changes in the input variables such as the effective porosity, bulk density, lithology, and the travel time of shear and Stoneley waves against the formation permeability.

## Modeling strategies

### EAs

#### PSO

Inspired by the social life of creatures such as birds and insects, a population-centric algorithm called PSO was created to optimize problems. Initially, random answers, known as particles, are generated^42^. Then, by updating the first generation of problem answers, the optimal answer is obtained^43^. Any possible answer by flying in the space of the problem seeks to achieve the optimal answer^44^. In the PSO method, a cluster of particles is called swarm. In a multidimensional space, particles of a sample population fly to a new location. The particles in this category are affected by the extent to which they are successful in locating their target neighborhood^45^. So, they work together in a neighborhood and society as a whole. Based on this external feature, a neighborhood with specific characteristics can be assigned to a particle. Neighborhoods can be divided into three categories: physical, social, and queen. Interested readers are advised to refer to Sharma and Onwubolu^45^ for more information. Assume that the parameters *g*_*best, d*_, *P*_*best*_, _*id*_, *v*_*i*_*(t)*, and *x*_*i*_*(t)*, respectively, indicate the best global location, the best previous location obtained by the *i-th* particle, the velocity vector of *i-th* particle in the *t-th* trial and error, and the location of *i-th* particles in *t-th* trial and error. Therefore, the velocity vector at each step of the iteration is corrected as follows^44, 46^:

1 $$v_{id} (1 + t) = v_{id} (t)w - c_{1} r_{1} (x_{id} (t) - p_{best,id} (t)) - c_{2} r_{2} (x_{id} (t) - g_{best,d} (t)), \, d = 1,2,...,D$$

In which, $c_{1}$ and $c_{2}$ represents the learning rate, $w$ shows the weight of the inertia, and $r_{1}$ and $r_{2}$ stand for the random numbers between zero and one^47^.

According to Eq. (1), velocity vector consists of three main components: inertial, cognitive, and social modules^42, 44^. To obtain the new particle location, the previous particle location must be added to its modified velocity vector according to the following equation^44^:

2 $$x_{id} (1 + t) = v_{id} (t + 1) + x_{id} (t), \, d = 1,2,...,D$$

where, *v*_*id*_, *x*_*id*_ and *t* indicate the velocity vector of *i-th* particle, the location of *i-th* particles, and the number of trial and error. More information about the theoretical description of the PSO can be found in open literature^48–52^.

#### GA

GA is classified as an evolutionary optimization method that can solve complex phenomena without any formulation of the objective function. In this method, random answers are generated by a random selection of chromosomes. In GA, numerous operators, such as mutation, reproduction, and crossover, are used to produce the best response from preliminary chromosomes. Using the Mutation Factor (MF) and Crossover Factor (CF), a parameter called Offspring Production (OP) is created to describe the binary selection of chromosomes in terms of probability changes between zero and one^53–55^. The detailed description of the GA strategy is depicted in the open literature^51, 52, 56–60^.

#### CSA

Modification of the Simulated Annealing (SA) algorithm leads to the development of a new method called Coupled SA (CSA), in which there is no significant reduction in its convergence rate. In order not to get into trouble of local optimal points, SA allows us to have a shift from initial to lower quality answers. During processing, the possibility of this displacement decreases. Therefore, the CSA algorithm was introduced to get rid of optima of local type and thus improve optimization. The most important difference between SA and CSA is the so-called parameter of the Acceptance Probability (AP). For the successful optimization of a divergent problem, Suykens and Vandewalle^61^ proved that by coupling the local optimization processes, more optimal solutions to the problem could be found^62^.

### Predictive techniques

#### ANFIS

For the first time, Jang^63^ combined fuzzy logic and artificial neural networks to develop a model called ANFIS. In this algorithm, the disadvantages of the previous models, namely fuzzy logic and neural networks, are eliminated. The ANFIS algorithm is categorized as a rule-based adaptive method so that these rules are generalized during the model training process^64^.

Using the IF–THEN fuzzy rule proposed by Takagi and Sugeno^65^, the relationship between inputs and outputs in ANFIS mathematical strategy is determined. The computational process in an ANFIS model with *z*_*1*_ and *z*_*2*_ as inputs of the model, and *f* as simulation output is as follows^64, 66–68^:

*Rule 1:*

3 $${\text{IF}}\,z_{1} \,{\text{is}}\,A_{1} \,{\text{and}}\,z_{2} \,{\text{is}}\,B_{1} ,{\text{ THEN}}\,f_{1} = r_{1} + p_{1} z_{1} + q_{1} z_{2}$$

*Rule 2:*

4 $${\text{IF}}\,z_{1} \,{\text{is}}\,A_{2} \,{\text{and}}\,z_{2} \,{\text{is}}\,B_{2} ,{\text{ THEN}}\,f_{2} = \, r_{2} + p_{2} z_{1} + q_{2} z_{2}$$

In general, the ANFIS mathematical strategy uses five internal computational layers^63^, which are as follows^63, 64, 66–68^:

*1. First layer: input nodes—*Each input node with the arbitrary index *i* is an adaptive node that follows the following rule:

5 $$O_{i,1} = \mu_{Ai} (z)\quad for \, i = 1,2 \ldots$$

In which, $O_{i,1}$ and $\mu_{Ai}$, in turn, represent the output of *i-th* node, and membership function of the *A*_*i*_ parameter. Typically, the following membership function, known as the Gaussian equation, is applied in calculations^64, 69^:

6 $$\mu_{{A_{i} }} (x) = e^{{ - \left( {\frac{{(x - c_{i} )^{2} }}{{2\sigma_{i}^{2} }}} \right)}} \quad for \, i = 1,2$$

*2. Second layer: rule nodes—*Each node in the second layer is considered as a fixed node that multiplies all the input signals and derives the result according to Eq. (7)^63, 64, 66–68^:

7 $$O_{2,i} = w_{i} = \mu_{{A_{i} }} (z) \times \mu_{{B_{i} }} (z)\quad for \, i = 1,2$$

*3. Third layer: normalization nodes—*At this stage, the weight factor is normalized by node *i*. To do this, the ratio of the *i*-weight node to the sum of the total weight nodes is calculated using the following formula^63, 64, 66–68^:

8 $$O_{3,i} = \overline{w}_{i} = \frac{{w_{i} }}{{w_{1} + w_{2} }}\quad for\,\,i = 1,2$$

where, the parameters $\overline{w}_{i}$ and $O_{3,i}$, respectively, show the normalized weight coefficient and the output of the third layer.

*4. Fourth layer: consequent nodes—*The signals in the fourth layer are separated according to the following equation^63, 64, 66–68^:

9 $$O_{4,i} = f_{i} \overline{w}_{i} = (r_{i} + q_{i} y + p_{i} x)\overline{w}_{i} \quad for \, i = 1,2$$

*5. Fifth layer: output nodes—*Each node in this layer calculates the final output of the model as a sum of all the received signals according to Eq. (10)^63, 64, 66–68^:

10 $$O_{5,i} = \sum\limits_{i} {\overline{w}_{i} } f_{i} = \frac{{\sum\nolimits_{i} {w_{i} f_{i} } }}{{\sum\nolimits_{i} {w_{i} } }} = final\,\,output,\,\,\,for \, i = 1,2$$

where, the parameters $\overline{w}_{i}$, $f_{i}$ and $O_{5,i}$, respectively, show the normalized weight coefficient, the simulation outputs and output of the fifth layer. In this study, the coefficients of the ANFIS model were tuned by using PSO optimization method. For more information, the interested readers are suggested to refer to the available literature^48, 70^.

#### Artificial neural network (ANN)

The ANN is one of the first generations of intelligent models for soft computing, which has numerous capabilities such as improving efficiency, adapting to environmental changes, and teaching/learning with an experience-oriented focus^71^. In this parallel distribution method, the simplest element is the neuron processor, which is connected to each other and organized in different layers. Two well-known types of ANN modeling are RBFNN and MLPNN. The MLPNN consists of three layers: input, hidden (intermediate), and output. There are a number of neurons in each layer. The number of neurons in the hidden layer is determined by an optimization method. The relationships governing the neural structure of MLPNN are related to the parameters of the parallel structure problems, and the model training process is performed as structural interconnections. To achieve the best MLPNN structure, inter-neural connections must be established using appropriate optimization algorithms^72^.

**Figure 2:**
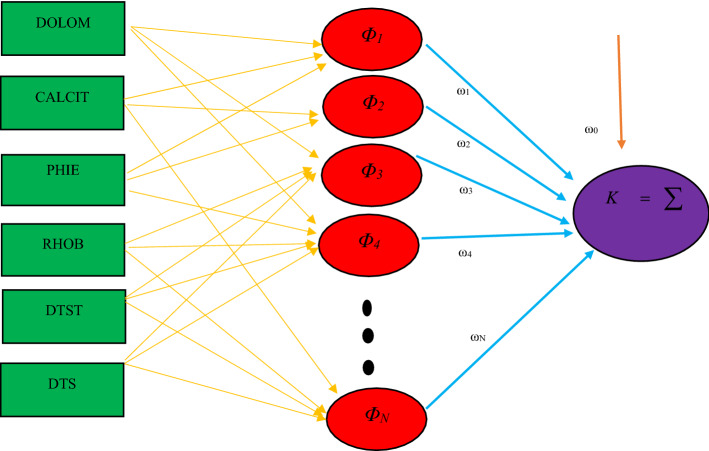
Schematic structure of the RBFNN modeling.

In RBFNN modeling, computational process design is simpler than MLPNN approach. For patterns where the MLPNN is not applicable, the RBFNN structure can provide very good feedback from their modeling^73^. Accordingly, RBFNN can be classified as a feed-forward neural network method that has been developed according to the iterative function approximation network and local base function. Due to the faster training process and the simpler structure that RBFNN has than MLPNN, the RBFNN is more popular and preferred in simulation^74^. The RBFNN can provide an ideal answer to any problem related to continuous functions with multiple inputs/outputs in a limited range. The desired modeling accuracy will be achieved by minimizing the defined Objective Function (OF) because the optimal specifications of the regulator network include three linear coefficients. In the RBFNN structure, it uses Radial Basis Function (RBF) as an activation function in the middle layer for each node/neuron. Model parameters include the exact shape of RBF, distance, and scale center. In linear modeling, we are dealing with fixed parameters. Therefore, the best global answer can be obtained with weight coefficients compatible with the minimum error^75^. Schematic structure of the RBFNN modeling is shown in Fig. 2. More information about the theoretical description of the MLPNN and RBFNN can be found in the open literature^76–78^.

#### LSSVM

A supervised learning method called support vector machine (SVM) was first developed by Vapnik^79^ based on statistical learning theory. This numerical structure can be used for a number of issues related to classification and regression. In addition to the many benefits of SVM, its calculations include solving several quadratic equations. This drawback of SVM is solved by introducing a newer SVM version called the LSSVM method. Instead of quadratic programming, linear equations that lead to simplification of the optimization process, are used in the LSSVM strategy^80^. Assume *(x*_*i*_*,y*_*i*_*)*_*n*_ be a dataset with size *n*, *x*_*i*_ shows input variable and *y*_*i*_ is output parameters. The LSSVM method can now be used to calculate any function. The linear regression function can be expressed as follows^81^:

11 $$f(x) = \omega .\phi (x) + b$$

As *ω* represents the weight vector, *b* represents the constant coefficient and *φ(x)* refers to a nonlinear equation such as sigmoid, linear, radial basis, and polynomial function. To determine the optimal values of *b* and *ω*, the total value of the parameters indicating complexity and empirical risk must be minimized according to the following equation^82^:

12 $$\min \frac{1}{2}\left\| \omega \right\|^{2} + C\sum\limits_{i = 1}^{n} {(\xi_{i} + \xi_{i}^{*} )}$$

13 $$\begin{gathered} S.t.\left\{ {y_{i} - \left\langle {\omega ,\phi (x_{i} )} \right\rangle } \right. - b \le \varepsilon + \xi_{i} \hfill \\ \left\langle {\omega ,\phi (x_{i} )} \right\rangle + b - y_{i} \le \varepsilon + \xi_{i}^{*} \hfill \\ \xi_{i} ,\xi_{i}^{*} \ge 0 \hfill \\ \end{gathered}$$

The loss function independent of *ε* is shown in the following equation:

14 $$L_{\varepsilon } (f(x_{i} ),y_{i} ) = \left\{ \begin{gathered} 0 \hfill \\ \left| {f(x_{i} ) - y_{i} } \right| - \varepsilon \hfill \\ \end{gathered} \right.\left. \begin{gathered} for\left| {f(x_{i} ) - y_{i} } \right| < \varepsilon \hfill \\ otherwise \hfill \\ \end{gathered} \right\}$$

To optimize, the Lagrangian saddle point will be obtained using the problem presented in Eq. (12) and its limitations shown in Eq. (13) as follows:

15 $$\begin{aligned}{L_{SVM}} & = 0.5 \times {\left\| \omega \right\|^2} + C\sum\limits_{i = 1}^n {(\xi _i^* + {\xi _i})} - \sum\limits_{i = 1}^n {({\xi _i} + \varepsilon - {y_i} + \left\langle {\omega ,\phi ({x_i})} \right\rangle + b) \times {\alpha _i}} \nonumber\\ & \quad - \sum\limits_{i = 1}^n {(\xi _i^* + \varepsilon + {y_i} - \left\langle {\omega ,\phi ({x_i})} \right\rangle - b)} \times \alpha _i^* - \sum\limits_{i = 1}^n {(\xi _i^*\eta _i^* + {\xi _i}{\eta _i})}\end{aligned}$$

Symbols $\alpha_{i}^{*} ,\alpha_{i} ,\eta_{i}^{*} ,\eta_{i}$ are Lagrangian multipliers. To create a set of optimization equations, a differentiation from the above equation is taken in terms of *b*, $\xi_{i}$, $\omega$ and $\xi_{i}^{*}$. As a result, the following set of equations is established:

16 $$\left\{ \begin{gathered} \frac{{\partial L_{SVM} }}{\partial \omega } = 0 \Rightarrow \omega = - \sum\limits_{i = 1}^{n} {\phi (x_{i} )(\alpha_{i}^{*} - \alpha_{i} )} \hfill \\ \frac{{\partial L_{SVM} }}{\partial b} = 0 \Rightarrow \sum\limits_{i = 1}^{n} {(\alpha_{i}^{*} - \alpha_{i} ) = 0} \hfill \\ \frac{{\partial L_{SVM} }}{{\partial \xi_{i} }} = 0 \Rightarrow C - \eta_{i} - \alpha_{i} = 0 \hfill \\ \frac{{\partial L_{SVM} }}{\partial b} = 0 \Rightarrow C - \eta_{i}^{*} - \alpha_{i}^{*} = 0 \hfill \\ \end{gathered} \right.$$

One of the optimization conditions is the validation of the following relationship:

17 $$\begin{gathered} \mathop {Max}\limits_{{\alpha ,\alpha^{*} }} (Q) = - \frac{1}{2}\sum\limits_{i,j = 1}^{n} {(\alpha_{i}^{*} - \alpha_{i} )} (\alpha_{j}^{*} - \alpha_{j} )\left\langle {\phi (x_{i} ),\phi (x_{j} )} \right\rangle - \varepsilon \sum\limits_{i = 1}^{n} {(\alpha_{i}^{*} + \alpha_{i} )} + \hfill \\ \sum\limits_{i = 1}^{n} {y_{i} } (\alpha_{i}^{*} + \alpha_{i} ) \hfill \\ \end{gathered}$$

18 $$S.t.\left\{ \begin{gathered} \sum\limits_{i = 1}^{n} {(\alpha_{i}^{*} + \alpha_{i} ) = 0} \hfill \\ \alpha_{i}^{*} ,\alpha_{i} \in \left[ {0,C} \right] \hfill \\ \end{gathered} \right.$$

The kernel function is written as follows:

19 $$K(x_{i} ,x_{j} ) = \phi (x_{i} )^{T} \phi (x_{j} )$$

So, the SVM problem is designed by the following equation^83^:

20 $$f(x) = \sum\limits_{i = 1}^{n} {(\alpha_{i} - \alpha_{i}^{*} )} K(x,x_{i} ) + b$$

Complex SVM mathematics has reduced the popularity of this model. This is due to the limitations in optimizing the SVM algorithm. Therefore, by using linear programming, this shortcoming in LSSVM has been eliminated^84^. For more details about the theoretical descriptions of LSSVM, numerous researches are available the literature^50, 51, 56, 78^.

#### CMIS

**Figure 3:**
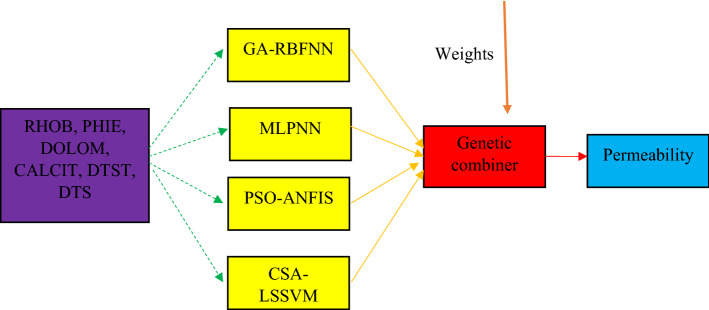
Structure and performance of the CMIS model.

CMIS, which was first established by Nilsson^85^ and then developed as a result of the work of Haykin and Network^86^, is an integration system from several other intelligent methods. Figure 3 shows the structure and functional mechanism of the developed CMIS algorithm in this study. Therefore, the CMIS output can be displayed as follows:

21 $$y_{CMIS}^{i} = ay_{MLPNN}^{i} + by_{RBFNN}^{i} + cy_{LSSVM}^{i} + dy_{ANFIS}^{i} + e$$

where, *y* indicates the prediction of each expert system. The coefficients *a*, *b*, *c*, *d* and *e* are equal to 0.6969, 0.7086, 0.0763, 0.3606, and − 0.1495, correspondingly. These coefficients are optimized by the GA method.

### Model development

In front of dense formations, the Stoneley wave velocity is essentially influenced by the formations and wellbore fluid characteristics. In front of the porous intervals, the shear slowness increases and the Stoneley wave slowness is affected by the formation shear modulus and bulk fluid modulus. At low frequencies, the following equation can be written^23^:

22 $$\frac{1}{{V_{st} }} = \sqrt {\rho_{f} \left( {\frac{1}{G} + \frac{1}{{K_{f} }}} \right)}$$

where, $V_{st}$ is the velocity of the Stoneley wave (in ft/μs), $\rho_{f}$ shows the borehole fluid density (in g/cc), *G* stands for the formation shear modulus and $K_{f}$ indicates the bulk fluid modulus. For rocks with pores, the shear modulus and bulky modulus are only equivalent values^87^. This equation is valid and correct in a formation with zero permeability that the Stoneley slowdown is only affected by the elastic properties of the well and formation. In front of the permeable zone, the behavior of the Stoneley wave is altered by fluid displacement. As the pore fluid can move relative to the solid matrix, this interaction lowers the energy and velocity of the Stoneley wave. Due to the higher formation permeability or mud fluidity, more Stoneley energy is reduced, and as a result, the wave slows down. Equation (22) links Stoneley slowness to the bulk formation density and shear slowness in the impermeable formations^23^. The following formula can be written in front of the permeable zones:

23 $$DTST^{\exp .} = DTST^{pred.} + f\left( {Mobility} \right)$$

where, $DTST^{\exp .}$ and $DTST^{pred.}$ represent, in turn, the measured and calculated Stoneley slowness. Having mud slowness and mud density, $DTST^{pred.}$ can be obtained from Eq. (34) theoretically. Equation (22) is low-frequency estimate of Stoneley wave slowness, which is obtained by simplifying more complex relationships. As a result, tuning the abovementioned parameters existing in Eq. (22) is more reliable instead of direct measurement of the mud property. Equation (22) can be re-arranged as follows:

24 $$DTST^{2} = \rho_{f} \frac{{DTS^{2} }}{{\rho_{b} }} + DT_{f}^{2}$$

in which, $DTST$, $DTS$, $DT_{f}$, $\rho_{f}$ and, $\rho_{b}$, respectively, stand for the Stoneley slowness (in μs/ft), the shear slowness (in μs/ft), mud slowness (in μs/ft), the mud density (in g/cc), and bulk formation density (in g/cc). According to Eq. (24), sketching $DTST^{2}$ versus $\frac{{DTS^{2} }}{{\rho_{b} }}$ in impermeable (zero porosity or shale) zones leads to a line with slope and intercept values equal to $\rho_{f}$ and $DT_{f}^{2}$, respectively. Thereby, Eq. (24) is determined clearly to be used for calculating $DTST$. Then, the Stoneley permeability index can be calculated by the following equation^14^:

25 $$KIST = \frac{{DTST^{\exp .} }}{{DTST^{pred.} }}$$

In the above equation, the Stoneley permeability index is shown by $KIST$, *DTST* indicates Stoneley slowness, and superscripts *exp.* and *pred.* are used to exhibit experimental and predicted values. This index indicates the amount of tortuosity of pore flowing channels better than their volume. Therefore, this index shows the specifications of hydraulic unit in the porous media. Considering Poisseulle model and Darcy's law, the following relationship can be presented to describe the permeability of the hydraulic unit^14^:

26 $$K = 1014FZI^{2} \left[ {\frac{{\phi_{e}^{3} }}{{\left( {1 - \phi_{e} } \right)^{2} }}} \right]$$

In Eq. (26), *K*, *FZI* and $\phi_{e}$ indicate absolute permeability (in md), flow zone indicator and effective porosity (in fraction). A linear relationship can be identified for *FZI* by using Eq. (26). In the impermeable zones, FZI becomes zero when the Stoneley permeability index reaches one; however, both *FZI* and *KIST* values are infinite in the highly permeable zones. Therefore, the following simple relationship can be put forward between *FZI* and *KIST*^8^:

27 $$FZI = IMF\left( {KIST - 1} \right)$$

In Eq. (27), *IMF* is known as the index matching factor. In this equation, IMF is the only tuning parameter for reaching a satisfactory match between the core permeability and the estimated permeability values by Eq. (26). Due to the fact that the grain modulus affects Stoneley wave slowness, the IMF could be calculated from the following equation^8^:

28 $$IMF = \sum {\left( {IMF_{i} V_{i} } \right)}$$

The symbols $IMF_{i}$ and $V_{i}$ indicate the index matching factor and volumetric composition of each mineral constituting the rock, correspondingly.

Based on the above theory and bulk of literature studies^8, 16, 18, 20–22, 24, 88^ for permeability calculation by Stoneley wave analysis, the following relationship can be proposed:

29 $$K = f\left( {RHOB,PHIE,DTST,DTS,DOLOM,CALCIT} \right)$$

where, $RHOB$, $PHIE$, $DTST$, $DTS$, $DOLOM$ and $CALCIT$ indicate bulk formation density, effective porosity, Stoneley wave slowness, shear wave slowness, and volumetric composition of dolomite and calcite in the formation lithology. Permeability is a tensor quantity, in which its determination is in need of anisotropy determination. For this goal, full waveforms are processed to determine anisotropy using borehole loggings. The joint study of shear wave elements, including both transverse and vertical components, and Stoneley wave velocity leads to the determination of waveform anisotropy. The main consequence of anisotropy calculation is the determination of permeability, pressure, and stress tensors. In this study, the inclusion of *DTST* and *DTS* leads to a more accurate determination of anisotropy in permeability calculation^89^. *DTST* considers the tube-wise wave propagation through the formation and borehole interface, although the *DTS* measures the shear-wise movement of the acoustic waves into the porous media. Therefore, the obtained permeability from Eq. (29) could be a proper representative for the rocks with pores as a bulk.

For optimizing the innovative mathematical strategies developed in this study, the well-known cost function, namely Root Mean Square Error (RMSE), is utilized as follows^37^:

30 $$RMSE = \left( {\frac{{\sum\limits_{i = 1}^{N} {\left( {K^{\exp .} \left( i \right) - K^{pred.} \left( i \right)} \right)}^{2} }}{N}} \right)^{\frac{1}{2}}$$

in which, $K^{\exp .}$, $K^{pred.}$ and *N* stand for experimental permeability, the predicted values by the smart mathematical strategies in this study, and the size of the dataset applied for modeling.

## Results and discussion

As already mentioned, four powerful models were used, including Adaptive Network-Based Fuzzy Inference System, Least Square Support Vector Machine, Multi-Layer Perceptron Neural Network, and Radial Base Function Neural Network. These models were integrated with some of the most important optimization methods available in the literature, such as Genetic Algorithms, Particle Swarm Optimization, and Coupled Simulated Annealing leading to the creation of more robust hybrid connectionist methods. These models include PSO-ANFIS, CSA-LSSVM, GA-RBFNN, and MLP. Afterward, by combining these four mathematical strategies, a stronger model called Committee Machine Intelligent System was developed.

To reasonably evaluate the performance of the methods developed in this study, several investigations were performed, including graphical methods such as cross-plot, contour map, outliers technique, and sensitivity analysis, and parametric methods such as Root Mean Square Error (RMSE), Average Absolute Deviation (AAD) and R-square parameters. To perform a point-by-point analysis of permeability estimation, the permeability profile was sketched along different zones to compare the performance of the models established in this study with the core permeability data and their potential in determining micro- and semi-filled fractures. Finally, the profile of the best model in this study was evaluated via one of the methods derived from the Nuclear Magnetic Resonance (NMR) for estimating permeability.

The adjustable parameters of the utilized smart methods studied here are reported in Table 1. These parameters have been calculated using the prementioned optimization methods that have led to the production of the least error in modeling the permeability based on the Stoneley wave. Table 2 reports the error of intelligent models for various subsets. The definitions of the errors are depicted as follows:

31 $$AAD = \frac{{\sum\limits_{i = 1}^{N} {\left| {K^{pred.} (i) - K^{\exp .} (i)} \right|} }}{N}$$

32 $$RMSE = \left( {\frac{{\sum\limits_{i = 1}^{N} {\left( {K^{\exp .} \left( i \right) - K^{pred.} \left( i \right)} \right)}^{2} }}{N}} \right)^{\frac{1}{2}}$$

33 $$R^{2} = 1 - \frac{{\sum\limits_{i = 1}^{N} {\left( {K^{pred.} \left( i \right) - K^{\exp .} \left( i \right)} \right)^{2} } }}{{\sum\limits_{i = 1}^{N} {\left( {K^{pred.} \left( i \right) - \overline{{K^{\exp .} \left( i \right)}} } \right)^{2} } }}$$

**Table 1:** Optimized setting parameters of the developed smart mathematical strategies in this study.

MLPNN		ANFIS	
Number of output neuron layer	1	Type	Value/comment
Number of hidden neuron layer	11	Number of clusters	15
Number of input neuron layer	6	Number of MF parameters	156
Optimization method	Genetic Algorithm	Membership Function	Gaussian
Output layer activation function	Logsig	Optimization method	PSO
Hidden layer activation function	Purelin	Number of data used for testing	2143
Number of max iterations	1500	Number of data used for training	410
Number of data used for testing	2143	Iteration	1500
Number of data used for training	410	Population size	73
Number of best epoch	90	C_1_	1
		C_2_	2

RBFNN		LSSVM	
Number of hidden neuron layer	90	Kernel function	RBF
Number of input neuron layer	8	γ	186,704.92
Hidden layer activation function	RBF	σ^2^	0.789001
Number of output neuron layer	1	Population size	73
Optimization method	GA	Number of data used for testing	2143
Output layer activation function	Linear	Number of data used for training	410
Number of data used for training	410	Optimization method	CSA
Number of population	50	Iteration	1500
Number of max iterations	30	**–**	**–**
Number of data used for testing	2143	**–**	**–**

**Table 2:** Statistical parameters of the developed smart mathematical strategies over the applied database for train, test and total subsets.

Model	Subset	R^2^	AAD	RMSE
CSA-LSSVM	Total	0.0049	5.1272	76.7163
	Train	0.0015	9.8329	143.4372
	Test	0.0093	4.2295	55.5273
MLPNN	Total	0.0022	5.4388	76.7712
	Train	0.0032	9.8625	143.3572
	Test	0.0029	4.5949	55.6568
GA-RBFNN	Total	0.8707	4.0462	31.0551
	Train	0.9990	1.9719	15.2574
	Test	0.7264	4.4419	33.2263
ANFIS	Total	0.0046	5.1815	76.7083
	Train	0.0017	9.8491	143.4120
	Test	0.0083	4.2911	55.5265
CMIS	Total	0.8726	3.7908	29.3918
	Train	0.9979	2.7634	31.8129
	Test	0.7322	3.9868	28.9069

in which, $N$, $K^{pred.}$ and $K^{\exp .}$ symbolize the number of datapoints used in the modeling process, the predicted, and actual rock permeability values, correspondingly. R^2^ parameter shows the degree of fitness of the predicted/calculated permeabilities compared to measured/target data. This statistical parameter varies from zero to unity indicating the poorest fitness (unreasoned relationship between input and output data) and the best fitness (reasonable and logical relationship between input and output data), respectively. AAD exhibits the mean value of the absolute error, in which the lower the AAD parameter results in the lower deviations from actual/target data. A zero value is desirable for AAD parameter. In addition, RMSE is also used to assess the model’s performance, which deals with the average of the square of the error value. The square root of the obtained value would be given as RMSE.

Based on Table 2, the total values of AAD and RMSE are, respectively, equal to 5.13 and 76.72 for CSA-LSSVM, 5.44 and 76.77 for MLPNN, 4.05 and 31.06 for GA-RBFNN, 5.18 and 76.71 for PSO-ANFIS, and 3.79 and 29.39 for CMIS. Based on this, it can be decided that the CMIS and GA-RBF models, in turn, provide the lowest prediction errors in computing the permeability of the carbonate reservoir rock. Also, the error parameters in all smart and deep learning models in both test and learning phases approximately have the same orders of magnitude, which confirms that the over-training problem has not occurred in this study. It is crystal clear that the MLPNN gives the lowest prediction accuracy with the highest AAD and RMSE values, and the smallest amount of R^2^. In other words, the following order is established amongst the accuracy of the proposed models:

$${\text{CMIS }} > {\text{ GA}} - {\text{RBFNN }} > {\text{ CSA}} - {\text{LSSVM }} > {\text{ PSO}} - {\text{ANFIS }} > {\text{ MLPNN}}$$

**Figure 4:**
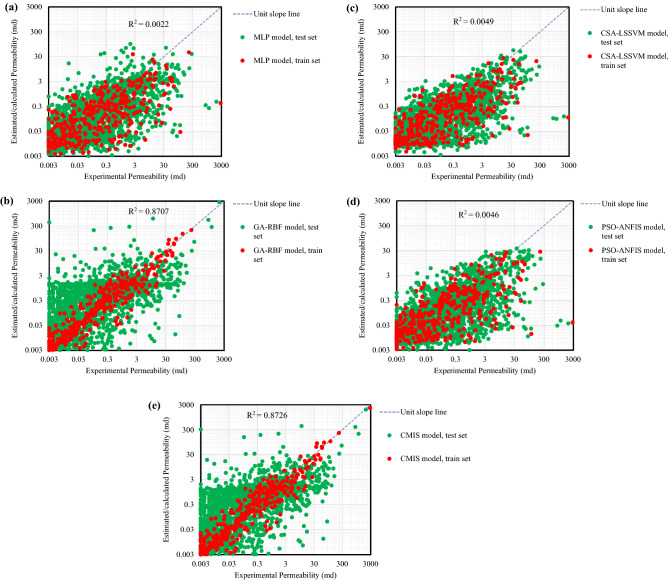
Estimated permeability by adverse smart mathematical strategies: (**a**) MLP model; (**b**) GA-RBF model; (**c**) CSA-LSSVM model; (**d**) PSO-ANFIS model; (**e**) CMIS model.

For comparing the measured core permeabilities with the estimated values by different machine learning techniques suggested here, the so-called parity diagrams are illustrated in Fig. 4. According to this figure, the closer the data cloud is to the "Y = X" line, the more precise the model is to estimate the target parameter. This can be illustrated by the R^2^, which is clearly shown in Fig. 4. The closer the R^2^ value to unity leads to the greater accuracy of the modeling. As can be seen, for CMIS and GA-RBFNN models, correspondingly, the highest proximity of the data cloud to line "Y = X" is observed. The values of the R^2^ for CMIS and GA-RBFNN models are 0.8726 and 0.8707, respectively.

**Figure 5:**
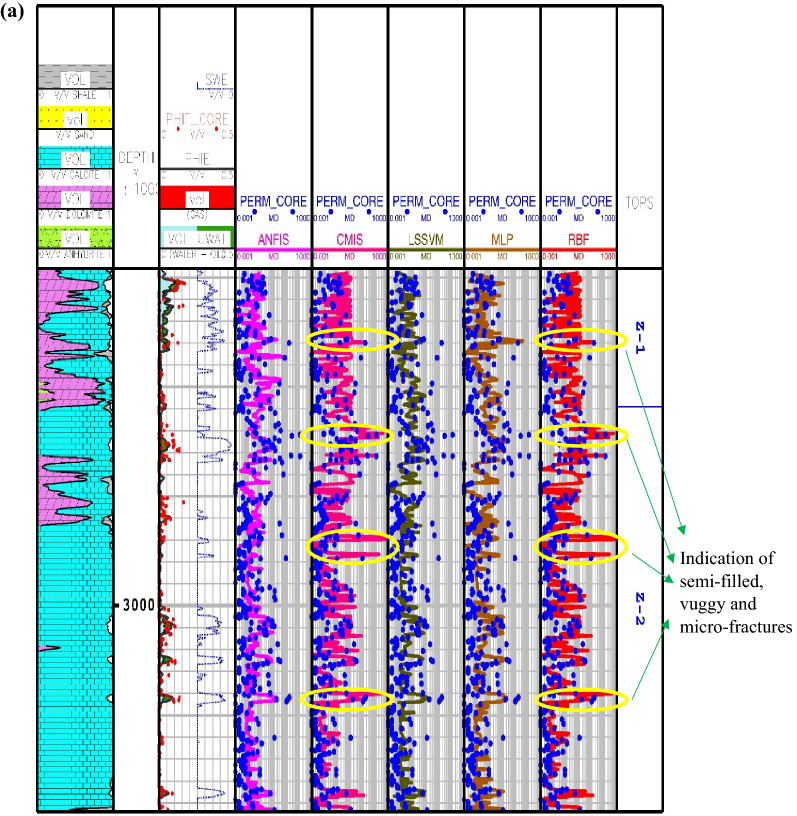

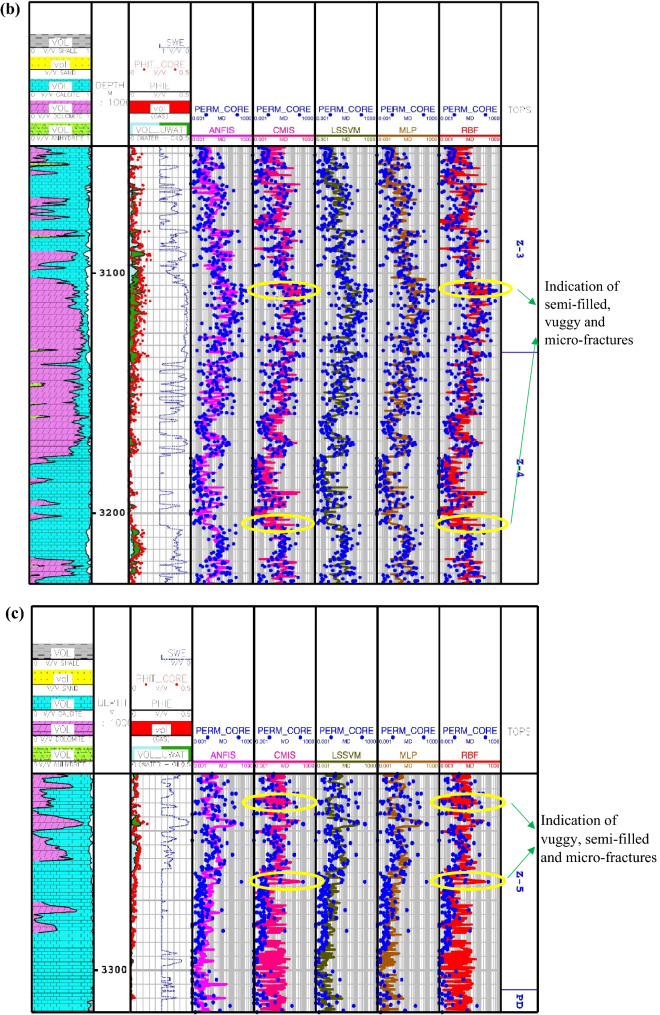
Comparison of the permeability derived from smart mathematical strategy with the core permeability data: (**a**) from Asmari zone 1 to zone 2; (**b**) from Asmari zone 3 to zone 4; (**c**) from Asmari zone 5 to top of PD formation (SWE: effective water saturation; PHIE: effective porosity; PHIT_CORE: core porosity; PERM_CORE: core permeability; TOPS: tops of geological zonation; PD: Pabdeh formation; VOL: volumetric composition of each mineral).

The results of the calculated permeations for the proposed smart methods are shown in Fig. 5 through Asmari formation zone 1 to 5 and top of Pabdeh formation. In this carbonate reservoir, the lithology is mainly made of lime and dolomite. As obviously shown, the core porosity data have a good match with porosity log obtained from petrophysical interpretation. Also, the porosity mainly varies between 5 and 10%, which confirms that the studied carbonate formation is an unconventional reservoir with fairly low porosity. As you can see, the results of the CMIS and GA-RBFNN are, in turn, much better compatible with core permeability data than other models. These two models, in addition to correct prediction of permeability trend, can also be used to detect vuggy, semi-filled and micro-fractures. These fracture indications are identified through the whole wellbore profile in Fig. 5. The available peaks in core permeability log at depths of 2960, 3020, 3107, 3150, 3205, 3227, 3247 and 3268 m give evidence to the claim that the GA-RBFNN and CMIS models have strong potential to successfully detect permeability changes in micro-fractures and semi-filled ones in the low-porosity carbonate formations.

Another available literature method for permeability prediction is derived from NMR logging. Two main techniques for permeability prediction have been developed namely Timur-Coates and Schlumberger-Doll-Research (SDR). Relaxation time method (T2) or SDR has been introduced by Kenyon et al.^90^ with the following formulation^23^:

34 $$K_{SDR} = c_{1} \left( {\phi_{NMR} } \right)^{{b_{1} }} \left( {T_{2,\log } } \right)^{{a_{1} }}$$

where, $K_{SDR}$ represents SDR permeability (in md), $\phi_{NMR}$ is the overall porosity of the NMR log (in percentage), and $T_{2,\log }$ shows the average logarithmic representation of the T2 distribution time (in ms). In Eq. (34), the values of experimental stability in carbonate formations are equal to *a*_*1*_ = 2, *b*_*1*_ = 4, *c*_*1*_ = 0.4^90^. The second model for permeability is known as the free fluid model, which was developed by Timur^91^ with the following formula^23^:

35 $$K_{TIM} = c_{2} 10^{4} \left( {\phi_{NMR} } \right)^{{b_{2} }} \left( {\frac{FFI}{{BFV}}} \right)^{{a_{2} }}$$

**Figure 6:**
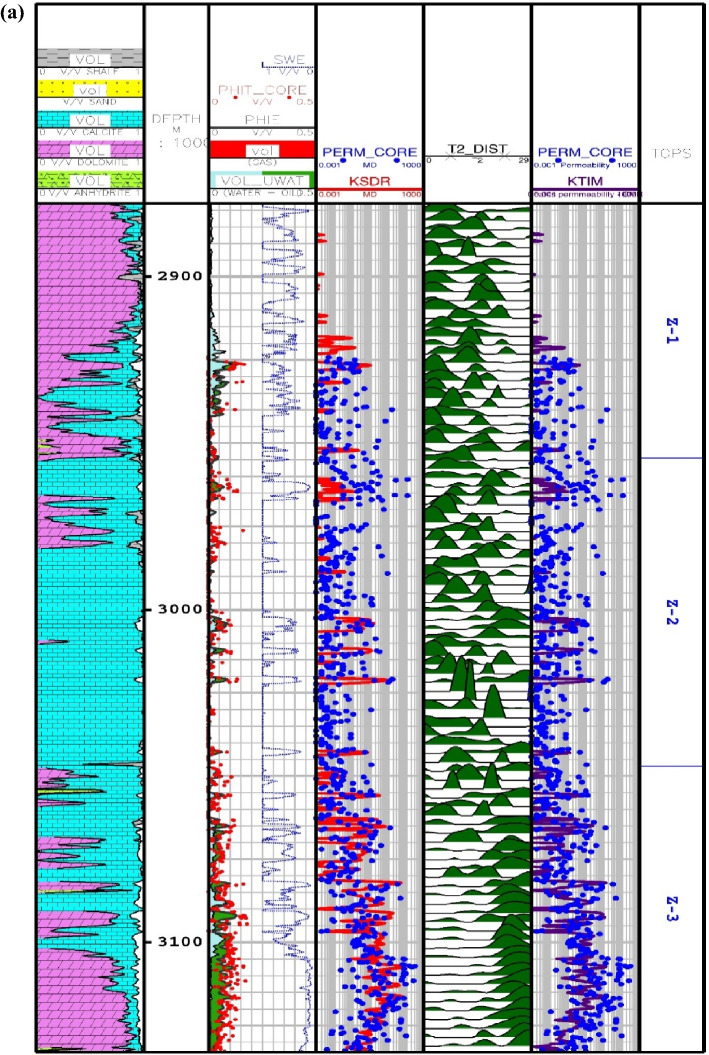

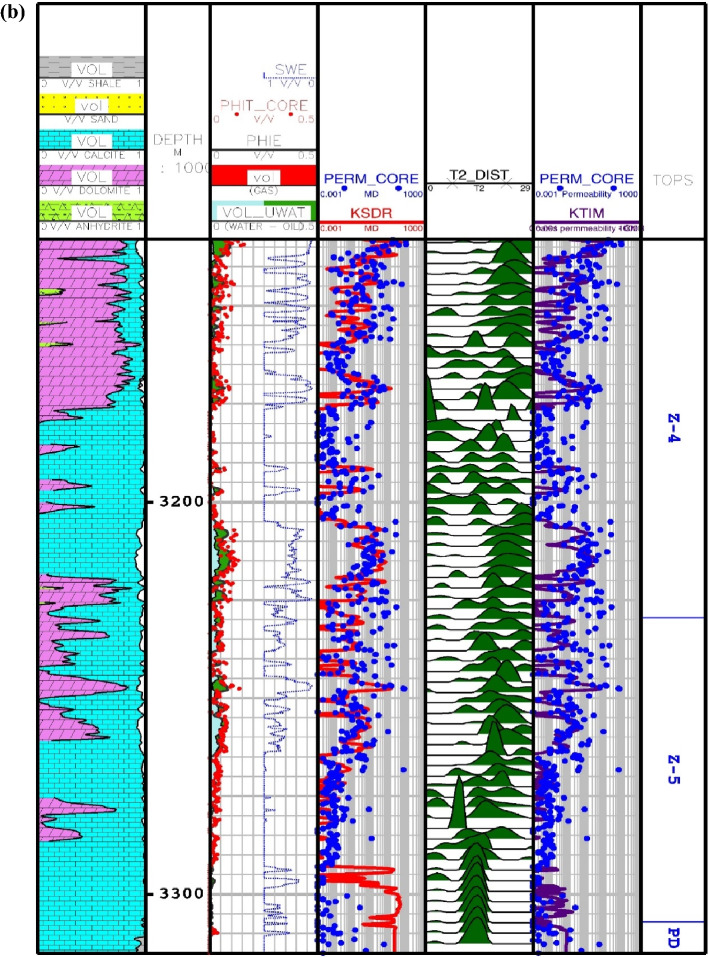
Comparison of the permeability derived from NMR logs with the core permeability data: (**a**) from Asmari zone 1 to zone 3; (**b**) from Asmari zone 4 to top of PD formation (PD: Pabdeh formation; T2_DIST: distribution of T2 relaxation time; KSDR: SDR permeability; KTIM: Timur permeability).

**Figure 7:**
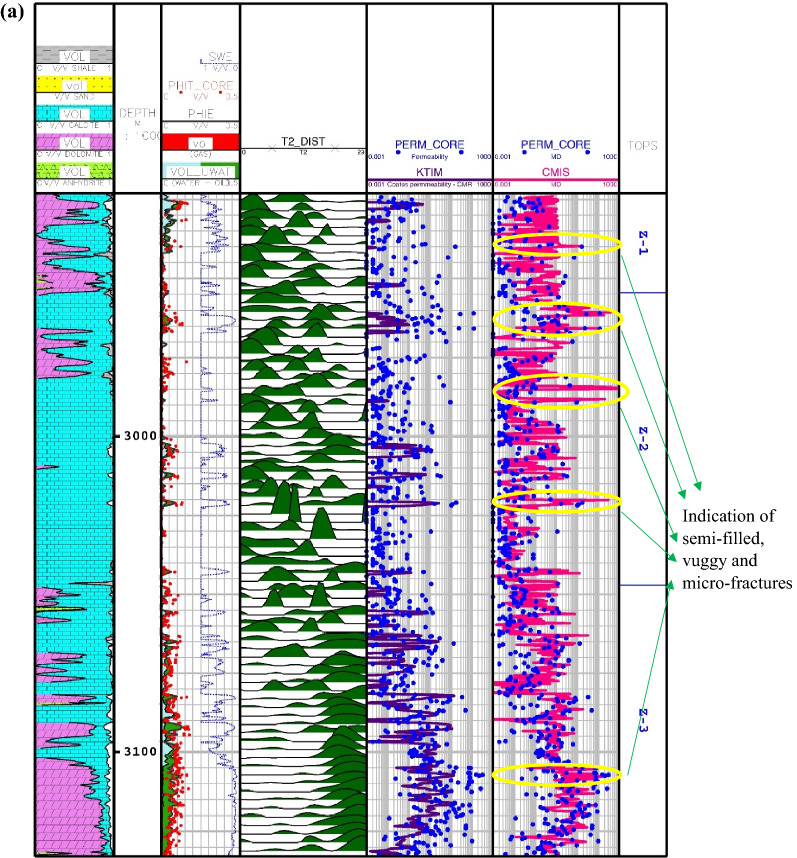

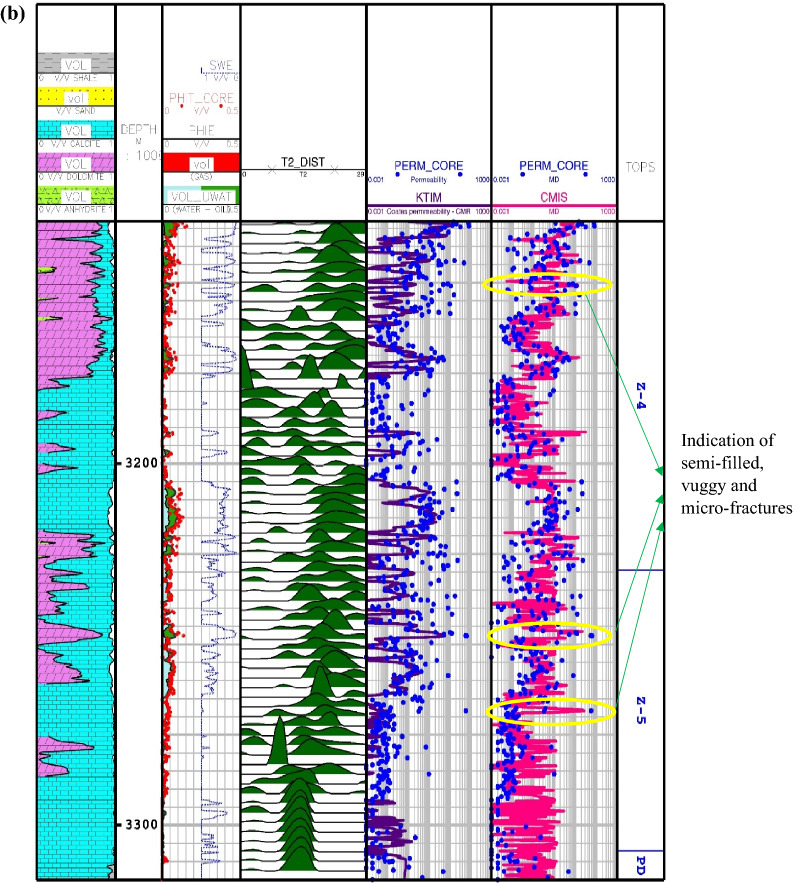
Comparison of the CMIS smart strategy developed in this study with Timur-Coates NMR model: (**a**) from Asmari zone 1 to zone 3; (**b**) from Asmari zone 4 to top of PD formation.

**Figure 8:**
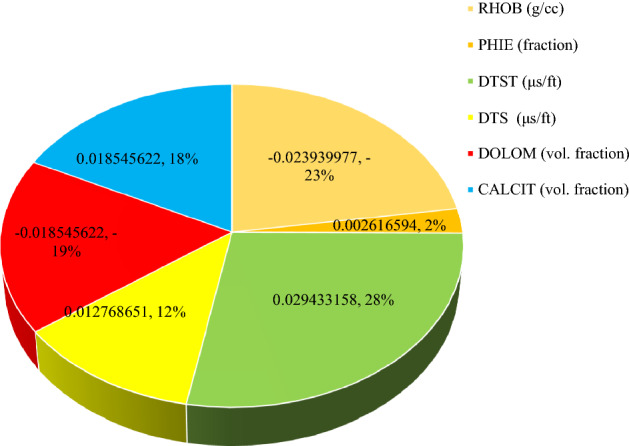
Sensitivity analysis of the variables considered for modeling Stoneley permeability analysis using CMIS smart mathematical strategy as the best model.

**Figure 9:**
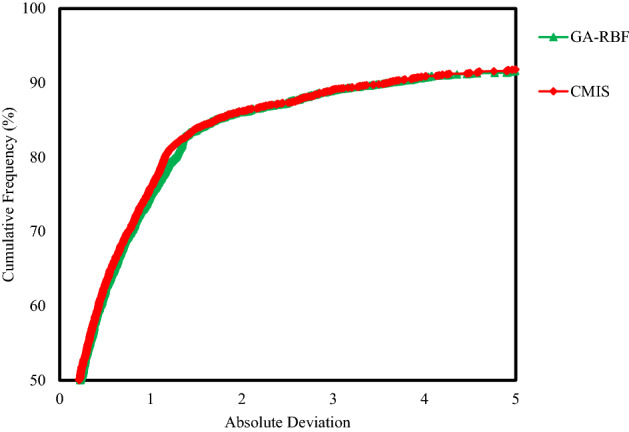
Cumulative frequency versus the absolute deviation for GA-RBF and CMIS smart mathematical strategies.

**Figure 10:**
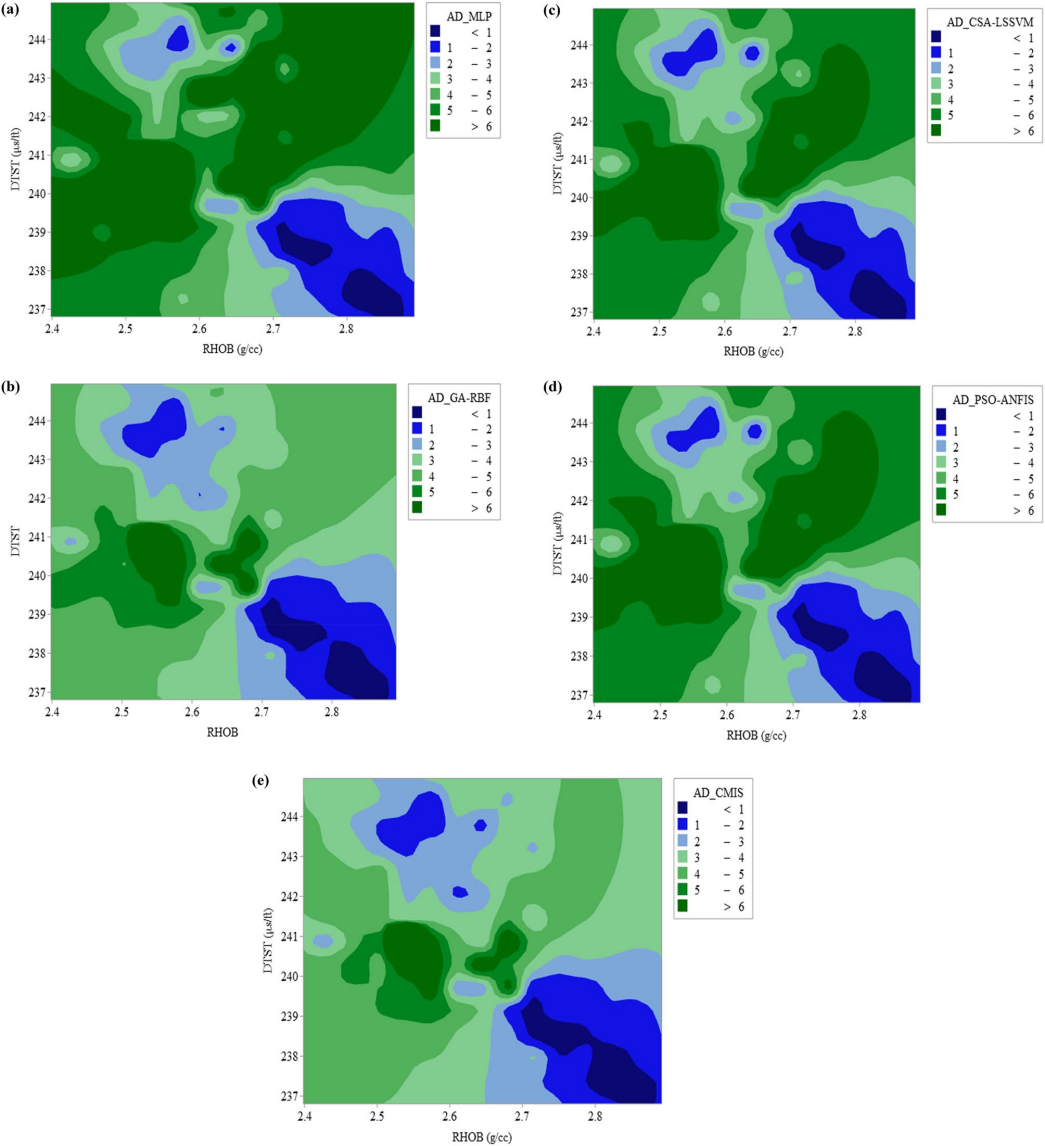
Variation of estimation error for extended smart mathematical strategies based on Stoneley wave analysis in this study: (**a**) MLPNN; (**b**) GA-RBFNN; (**c**) CSA-LSSVM; (**d**) PSO-ANFIS; (**e**) CMIS.

In Eq. (35), $K_{SDR}$, $\phi_{NMR}$, $FFI$ and $BFV$ represent, in turn, the SDR permeability (in md), the total porosity obtained from the NMR log (in percentage), and the free fluid and the bound fluid volume. In Eq. (35), the empirical coefficients in carbonate formations are equal to *a*_*2*_ = 2, *b*_*2*_ = 4, *c*_*2*_ = 0.1^91^. Figure 6 indicates the NMR permeability profiles attained by SDR (i.e., Eq. (34)) and Timur-Coates (i.e., Eq. (35)) throughout Amari formation zone 1 to 5 and top of Pabdeh formation. First of all, the distribution of T2 or relaxation time log shows a good relative correlation with the porosity logs obtained from both core data and petrophysical evaluation. The permeability results from SDR and Timur-Coates models are fairly matched to the core permeability data. In Pabdeh formation, the SDR permeability profile is highly disturbed by borehole washout; therefore, only Timur-Coates model (i.e., Eq. (35)) is used for the rest of analysis. For checking the applicability of the best smart method suggested in this study, the CMIS approach is compared with Timur-Coates model (i.e., Eq. (35)), as shown in Fig. 7. Alongside the entire wellbore, the CMIS permeability profile shows highly better agreement with the core permeability log. NMR method (i.e., Eq. (35)) poorly matches with measured permeability data and large deviations can be observed from the actual data; however, CMIS method accurately follows the main permeability variations in all zones. Furthermore, in very high permeable intervals indicating semi-filled, micro- and vuggy fractures, CMIS predicted permeability well matches the actual data, even though NMR log predominantly ignores those fracture indicating layers. Several pieces of evidence for these fracture indications are shown in Fig. 7. The main reason for this great consistency of the CMIS model predictions with the real data is the presence of Stoneley slowness log as one of the inputs to the CMIS model. The Stoneley wave propagates cylindrically in the interface of the wellbore and the formation, which is why it is also called a tube wave. In permeable and cracked zones, the pore spaces in the rock increase and cause the Stoneley wave to be trapped in the caves and fractures. Therefore, the energy of the Stoneley wave decreases, which leads to an increase in the travel time or slowness of the Stoneley wave. Figure 8 indicates the outcomes of the sensitivity analysis via Pearson’s technique^92^. As can be seen in this figure, to determine the exact amount of effect of each variable on the output prediction, a normalized value between -1 and + 1 is calculated as the impact value of that variable, which is obtained by the following formula^92^:

36 $$r = \frac{{\sum\limits_{i = 1}^{n} {\left( {I_{k,i} - \overline{{I_{k} }} } \right)\left( {O_{i} - \overline{O} } \right)} }}{{\sqrt {\sum\limits_{i = 1}^{n} {\left( {I_{k,i} - \overline{{I_{k} }} } \right)^{2} \sum\limits_{i = 1}^{n} {\left( {O_{i} - \overline{O} } \right)^{2} } } } }}$$

where, $I_{k,i}$, *n*, $O_{i}$, $\overline{O}$ and $\overline{{I_{k} }}$ represent i-th input value of the k-th input parameter, the number of the dataset, i-th output value, mean value for output parameter and, mean value for the k-th input, respectively. In this figure, the highest and lowest impact values are attributed, respectively, to the Stoneley wave slowness and effective porosity. Besides, the slowness of Stoneley and shear waves, effective porosity and volume fraction of calcite in the reservoir rock have positive effects on the permeability prediction. In Fig. 9, cumulative frequency against the Absolute Deviation (AD) is drawn for GA-RBFNN and CMIS. The higher the cumulative frequency diagram at the same error value results in more precise model. For instance, when the absolute deviation is equal to 5.0, about 91.81% of CMIS estimates and 91.57% of GA-RBFNN predictions have errors equal or less than 5.0. Thus, the CMIS model gives more accurate permeability profile. Contour map analysis of AD variation versus Stoneley slowness and bulk formation density is exhibited in Fig. 10. Based on this diagram, the operational regions with less deviations (i.e., blue color and light green colors) cover wider portions of shown contour map for CMIS technique. In other words, larger areas of the PSO-ANFIS, CSA-LSSVM and MLPNN are allocated to AD values more than 4. Based on Fig. 10e, when 2.52 < RHOB < 2.58 g/cc, 2.62 < RHOB < 2.70 g/cc and 239 < DTST < 241.3 μs/ft, the highest deviation occurs in CMIS modeling.

**Figure 11:**
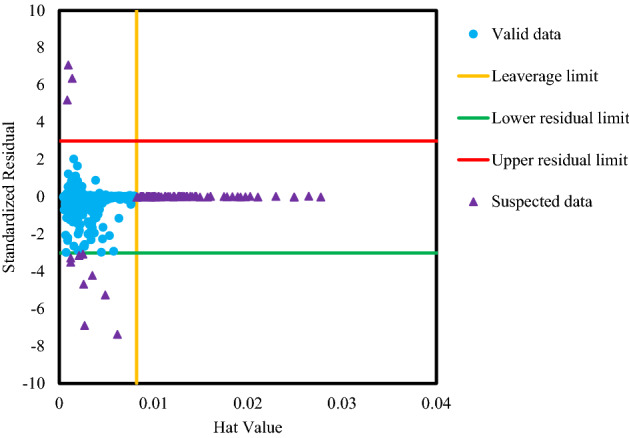
Outliers’ analysis of the developed CMIS model over the utilized database.

In the last analysis, a well-known plot named William’s diagrams is prepared as illustrated in Fig. 11. The degree of uncertainty in any database reduces the reliability of modeling conducted on that dataset. There are some intolerable errors in the system due to human and equipment errors during the log measurements and interpretations (i.e., lithology, effective porosity, bulk density and slowness of Stoneley and shear waves as input), and core measurements (i.e., permeability as output)^93, 94^. This part of the data, which behaves abnormally, is called outliers data. A method called Leverage method or William’s plot is used to detect these outliers’ data. In this graph, the standardized residuals (vertical axis) are plotted against the Hat value (horizontal axis). The following formula is used to calculate the Hat matrix^93, 94^:

37 $$H = X\left( {X^{T} X} \right)^{ - 1} X^{T}$$

where, Hat matrix, the input matrix with *m* rows (i.e., database size) and *n* columns (i.e., number of input data), and matrix transpose operation are shown with symbols *H*, *X* and superscript *T* correspondingly. The Hat values are recognized as the main diagonal of Hat matrix. Hat values have a critical restriction, which is defined as below^93, 94^:

38 $$H^{*} = \frac{{\left( {n + 1} \right) \times 3}}{m}$$

In above equation, $H^{*}$ shows the critical Hat value. In this study, $H^{*}$ is equal to 0.0082. In the shown outliers’ plot (see Fig. 11), a special region, namely applicability domain, is shown in which standardized residual varies between − 3.0 and + 3.0 and Hat value is less than 0.0082. Based on Fig. 11, about 3.7% of the database (94 data) are identified as suspected or outliers’ data. This means that the applied database here is valid and the CMIS model is truthful.

### Source of error

In this study, many factors affect the computation error. The database is derived from two different sources, including RCAL and wireline logging. These two measurements are performed separately at different times. Therefore, it is necessary to depth matched the data from RCAL with logging data, which is usually integrated with an error. The logging operation in oil and gas wells can also be affected by some environmental factors such as the mud type and its additives, the borehole diameter, the degree of washout, and tool quality and its calibration. RCAL measurements are influenced by adverse factors such as differences in well and laboratory conditions, sampling method, and human and the device errors. Therefore, there is an initial uncertainty that is noticeable in the database, which means that the error of the created model will never be less than a certain value; hence, the consistency between the predicted and the actual values is almost always not ideal. Clearly, it is difficult to implement a modeling study in the low-porosity carbonate formations, which are full of complex geological features.

## Conclusions

In this study, a number of hybrid machine learning methods, including Adoptive Network-Based Fuzzy Inference System (ANFIS) optimized with Particle Swarm Optimization (PSO), Least-Square Support Vector Machine (LSSVM) optimized with Coupled Simulated Annealing (CSA), Radial Basis Function Neural Network (RBFNN) optimized with Genetic Algorithm (GA), Multi-Layer Perceptron Neural Network (MLPNN), and Committee Machine Intelligent System (CMIS), were used to prediction formation permeability based on the guided theory of Stoneley wave propagation. For this purpose, a database including lithological data, bulk formation density, shear and Stoneley slowness, and effective porosity (as the input) and core permeability (as the output) were used in a low-porosity carbonate formation. Approximately, 16% of the database was used to train powerful algorithms, then the rest of the database was used to test and evaluate the models alongside the studied low-porosity carbonate formation. A number of statistical error parameters such as RMSE, AAD and R^2^, and diverse graphical analyses such as sensitivity analysis, cross plot and outliers’ data analysis were used to evaluate the models as accurately as possible. It is found that the slowness of the Stoneley wave has the greatest effect on the permeability prediction, even though the effective porosity has the least effect. Moreover, the CMIS model with R^2^ = 0.87 and RMSE = 29.39 offers the most accurate profile for permeability. Comparing the CMIS model with the permeability profile obtained from Timur-Coates model (i.e., NMR method) reveals that the intelligent model presented in this study has more accuracy and higher agreement with core data. Furthermore, features like vugs, micro-, and semi-filled fractures, which are mentioned in the Routine Core Analysis (RCAL) report, are easily detected by the CMIS model, while the Timur-Coates model has high weakness in diagnosing micro-fractures. According to the outliers’ analysis, it is proven that less than 4% of databank are outliers illustrating the database validity and the model reliability. Finally, it should be noted that the in-depth intelligent learning models developed in this work play an important role in assessing permeability profile in low-porosity carbonate formations, which can be used to improve the quality of relevant industrial studies and academic simulations through fluid flow studies in porous media.
